# Diet-microbiome coevolution: the core mechanism for semi-aquatic adaptation and cross-habitat niche coexistence of the web-footed shrew (*Nectogale elegans*)

**DOI:** 10.3389/fmicb.2025.1711143

**Published:** 2025-11-13

**Authors:** Jiayi Jiang, Sibei Zhou, Jia Song, Chongxie Xia, Xuexiang Yang, Kun Yang, Fengjun Li

**Affiliations:** 1 Institute of Ecology, China West Normal University, Nanchong, Sichuan, China; 2 College of Environmental Science and Engineering, China West Normal University, Nanchong, Sichuan, China; 3 Key Laboratory of Southwest China Wildlife Resources Conservation (Ministry of Education), China West Normal University, Nanchong, Sichuan, China

**Keywords:** *Nectogale elegans*, diet, gut microbiota, semi-aquatic adaptation, niche differentiation

## Abstract

The adaptation of mammals to semi-aquatic niches represents a pivotal ecological transition, in which the coevolution of dietary specialization and gut microbiome is regarded as playing a fundamental role. However, the general mechanisms that link these traits to survival across habitats remain insufficiently investigated, particularly in small mammals with high metabolic constraints. Using the web-footed shrew (*Nectogale elegans*), a rare small mammal with extreme semi-aquatic specialization, this study supplements the understanding of host-microbe symbiosis in the process of small mammals adapting to new ecosystem. To address how diet facilitates semi-aquatic adaptation, we integrated benthic community surveys and dietary DNA metabarcoding. Our results showed that the web-footed shrew primarily utilizes benthic macroinvertebrates (Diptera, Ephemeroptera, and Trichoptera), consistent with the composition of local benthic biomass, and supplemented by Cypriniformes fish. Comparative analysis of DNA metabarcoding with sympatric terrestrial rodents further revealed that semi-aquatic shrews achieve niche differentiation through two complementary mechanisms: habitat partitioning (aquatic vs. terrestrial) and trophic level differentiation (secondary consumers of invertebrates vs. consumers of plants). This discovery extends niche theory, demonstrating how habitat-specific resource utilization shapes trophic stratification. Compared to the terrestrial group, the gut microbiome of the semi-aquatic shrew exhibited significant differences in both microbiome composition and functional potential: dominance of Proteobacteria and Firmicutes, reduced abundances of carbohydrate-active enzymes (CAZymes), as well as selective enrichment of genes involved in fatty acid metabolism. These results reflect the high-fat, high-protein niche of semi-aquatic shrews. Additionally, the seasonal stability of the microbiome of the semi-aquatic shrew mirrors the consistency of benthic resources, and maintaining metabolic homeostasis is key to long-term adaptation to fluctuating environments. Overall, this study demonstrates a framework for semi-aquatic adaptation in small mammals: dietary specialization drives niche differentiation, which in turn selects for gut microbiome adaptation, optimizing habitat-specific resource utilization. This research underscores the role of diet-microbiome coevolution in enabling semi-aquatic adaptation, offering novel insights into ecological niche differentiation and specialization mechanisms in small mammals.

## 1 Introduction

The stark contrast between aquatic and terrestrial habitats presents a substantial challenge to semi-aquatic species, necessitating significant adjustments in lifestyle, physiological regulation, and behavioral patterns (Soininen et al., 2015). Semi-aquatic mammals, serving as transitional forms between aquatic and terrestrial lifestyles, exploit resources from both ecosystems, offering a distinctive perspective for studying the co-evolution of microbial communities and their hosts (Mengistu et al., 2022). Numerous current studies focus on the microbial composition of large and medium-sized semi-aquatic mammals [e.g., walruses (Couch et al., 2022), hippos (Masese et al., 2020), beavers (Gruninger et al., 2016), and otters (Guo et al., 2020)], while microbial studies in small semi-aquatic mammals remain underrepresented despite their unique ecological roles. Small mammals exhibit fast metabolic rates, high adaptability to confined spaces, and short reproductive cycles (Sun et al., 2019). Their rapid metabolic rates and confined habitats may drive distinct microbial adaptations, making them critical models for understanding host-microbe co-evolution under environmental constraints. The web-footed shrew (*Nectogale elegans*), a typical semi-aquatic small mammal, provides an ideal system to investigate the evolutionary pathways and ecological functions of host-associated microbial communities.

Among semi-aquatic small mammals, the web-footed shrew (*Nectogale elegans*) is highly specialized (Liu, 2019). It possesses a series of aquatic adaptive characteristics, including webbed feet, a trait showing significant convergent evolution with distant lineages such as beavers, platypuses, and otters (Dunstone et al., 1998). Its waterproof fur reduces heat loss in water, thereby maintaining a constant body temperature. A fringed tail enhances propulsion and steering during swimming, while reduced external ears minimize drag (Hutterer, 2005). These adaptations make it an excellent model for studying aquatic-terrestrial transitions. The species inhabits mountain streams at 900–4,600 meters altitude, distributed across the Himalayan region (Nepal, Tibet, Sikkim, Bhutan) and extending into western/central China and northern Myanmar (Smith and Xie, 2008). However, human activities, such as water resource development, are increasingly threatening its survival (Smith and Xie, 2008; Sharma et al., 2017). Key information about interactions between its dietary preferences, microbiota, and life history remains understudied, which is critical for conservation planning (Molur, 2016).

Diet, a fundamental driver of life activities, provides essential energy and nutrients while shaping gut microbial communities (Ferreira et al., 2022). Insectivorous mammals primarily obtain food by preying on insects (Ivanter et al., 2015). However, the physical differences between water and air (e.g., density, viscosity, and light propagation properties) present unique challenges for predation in aquatic environments. Visual identification of prey is cost-effective and operationally simple, but it requires expertise to distinguish subtle differences among plants, vertebrates, and insects, which necessitates extensive training and resources (Khanam et al., 2016). DNA metabarcoding, using universal primers, simultaneously processes multiple taxonomic groups across samples, significantly improving research efficiency and accuracy (Peng and Tao, 2023). By combining visual identification of potential prey resources with DNA metabarcoding analysis, this integrated approach is particularly valuable for species like the web-footed shrew, whose semi-aquatic lifestyle makes diet assessment inherently challenging (Hawlitschek et al., 2018).

From an ecological perspective, the gut serves as a unique microbial habitat (Maritan et al., 2024). Complex interactions exist between the gut microbial community and the host (Bletz et al., 2016), ranging from long-term symbiotic relationships to transient dynamics driven by external factors (Walter and Ley, 2011). These interactions form the basis for host-ecosystem interconnections and are critical for species adaptation to environmental conditions (Moeller and Sanders, 2020). The gut microbiota exhibits inherent heterogeneity and dynamic changes (Zoetendal et al., 2001), responding to host age, diet, and health status (Power et al., 2014; Hu et al., 2023). Existing research has well-established the pivotal role of microbial communities in processing dietary nutrients in small mammals (Holland, 2024). For instance, phylogenetic proximity often correlates with microbial and dietary similarities (Guo et al., 2023; Zhang et al., 2023), while distinct diet types drive divergent microbiota structures (Li et al., 2022), and seasonal dietary shifts further modulate gut microbial composition (Song et al., 2023). These findings highlight the intimate relationship between diet and gut microbiota, confirming that host genetics and environmental factors jointly maintain microbial diversity. This integration of genetic and environmental influences underscores the adaptive plasticity of gut microbiota in small mammals.

Niche construction theory refers to the process by which organisms actively modify their own or other species' niches (e.g., through foraging behavior or habitat modification) to improve their adaptive fitness (Odling-Smee et al., 2003). Significant differences in the types, quantities, and availability of food resources exist between aquatic and terrestrial habitats (Elser et al., 2000). These disparities influence food selection and energy intake in semi-aquatic shrews, thereby shaping microbiota structures and ecological adaptation. Given the ecological significance of these interactions, we aimed to investigate: (1) the diet composition of the web-footed shrew (*Nectogale elegans*) via field surveys and DNA metabarcoding sequencing; (2) gut microbial community differences between sympatric individuals in semi-aquatic vs. terrestrial habitats using metagenomic sequencing; (3) the effects of environmental changes, particularly seasonal variations, on gut microbiota composition and structure. Notably, no sympatric terrestrial shrew species were detected in our study area, which was confirmed by field surveys using live traps and snap traps at different seasons. And it is constrained our comparison to semi-aquatic shrews (*Nectogale elegans*) and sympatric terrestrial rodents. This absence may be related to the harsh arid-hot valley environment, which restricts the distribution of terrestrial shrews that require higher humidity. This research will provide insights into diet-microbiota dynamics of semi-aquatic mammals in natural ecosystems, contributing to conservation strategies by elucidating adaptive responses to environmental fluctuations.

## 2 Materials and methods

### 2.1 Study system

**Figure 1:**
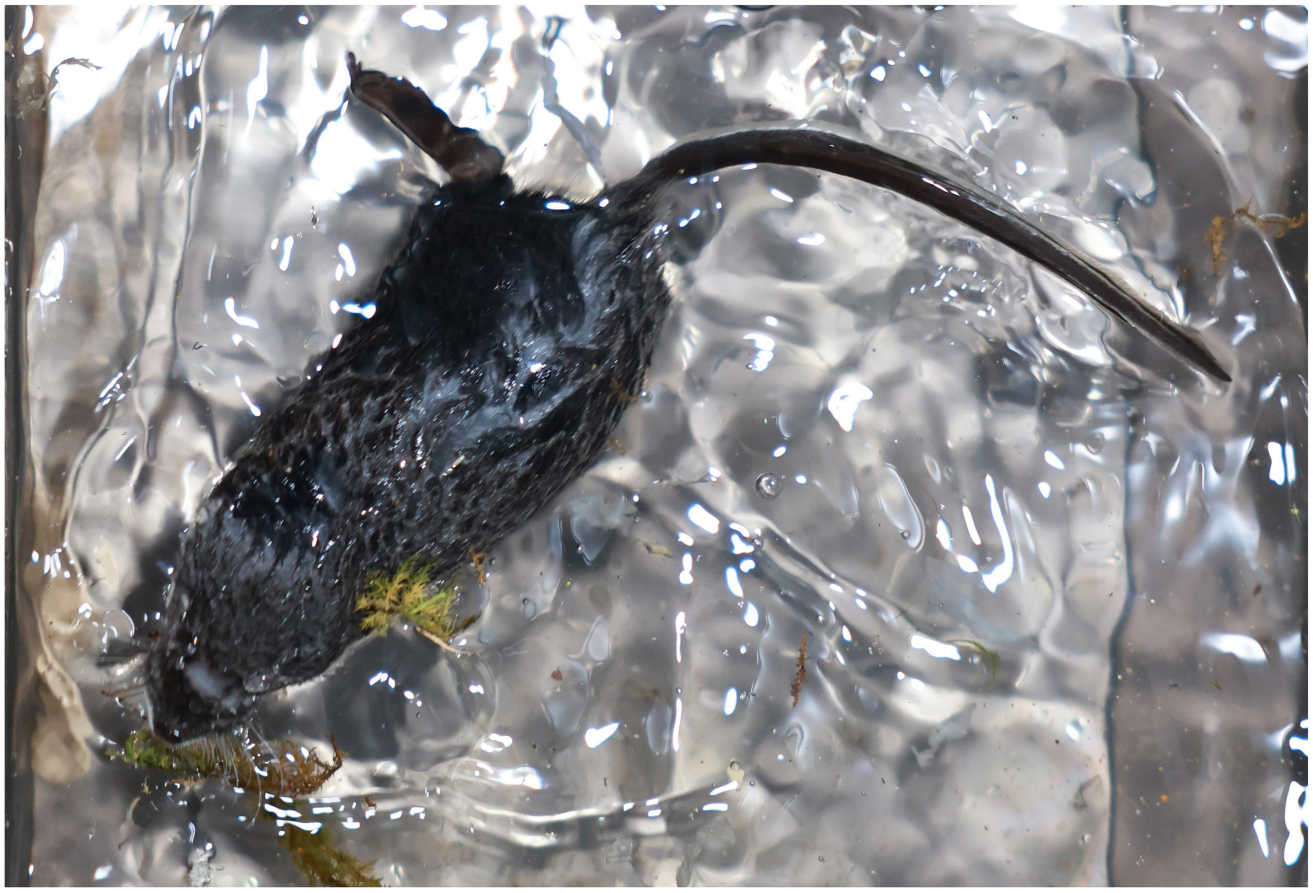
The web-footed shrew (*Nectogale elegans*).

Semi-aquatic and terrestrial species face contrasting abiotic and biotic conditions. In this study, we compared habitat-specific diet spectra and gut microbial community structures between the semi-aquatic web-footed shrew (*Nectogale elegans*, SE group, Figure 1) inhabiting tributaries of the Yalong River in Liangshan, Sichuan Province, China. Sympatric terrestrial rodents (TE group), including *Apodemus chevrieri, Rattus andamanensis*, and *Rattus tanezumi* (Supplementary Table 1), were sampled from the same riparian area adjacent to the aquatic foraging habitats of the web-footed shrew. The web-footed shrews (SE group) were captured using shrimp traps placed perpendicular to the current in shallow, slow-flowing stream sections, and no bait was used for shrew sampling. Sympatric terrestrial rodents (TE group) were captured using live traps baited with peanuts. A total of 14 individuals of SE group were collected from shrimp cages, and 6 samples of TE group were obtained via rat traps (Supplementary Table 1). Live animals were transported to the laboratory and housed individually in containers. They were humanely euthanized via cervical dislocation. Stomach and gut contents were then dissected, isolated, and placed into sterile tubes. Each sample was preserved in liquid nitrogen and transported for downstream analysis.

### 2.2 Food spectrum analysis

To identify the potential prey resources of the web-footed shrew, this study conducted visual identification of benthic invertebrates in the rivers of the sampling sites. Benthic invertebrates were collected using a Surber net during the dry and rainy seasons. Qualitative and quantitative benthic invertebrate samples were preserved in a 10% formaldehyde solution at room temperature. In the laboratory, samples were identified and counted under a stereomicroscope, then classified and weighed to calculate biomass. In addition, we used DNA metabarcoding technology to comprehensively analyze the food composition of the digestive tract contents (a mixture of stomach and gut contents). Mixed analysis of stomach contents and intestinal contents can cover the complete food spectrum from feeding to digestion, avoid information loss caused by the limitation of digestion stage in a single part, and improve the comprehensiveness of food composition detection. We used three sets of previously developed universal primers to amplify the DNA of vertebrates, invertebrates, and plants (Supplementary Table 2). We targeted the mitochondrial 16S mtDNA of vertebrates (Wang Z. et al., 2023), the mitochondrial COI mtDNA of invertebrates (Leray et al., 2013), and the chloroplast rbcL gene of plants (Hofreiter et al., 2000) for amplification. Notably, plant DNA analysis of the SE group was excluded because the library construction failed due to insufficient concentration, whereas TE group plant libraries remained unaffected. For diet DNA metabarcoding of the TE group, reads corresponding to peanuts were specifically filtered out during data preprocessing to eliminate bait-derived contamination.

### 2.3 DNA metabarcoding sequence and analysis

We employed the OBITools (Boyer et al., 2016) program to filter the sequences. The Obigrep command was used to remove sequences with a quality score of less than 40 and those shorter than 80 base pairs (bp) in the entire dataset. The Obiclean command was utilized to detect and discard PCR and sequencing errors. Subsequently, we used fastx_uniques in Usearch (Zhou et al., 2024) to remove duplicate sequences. The cluster_otus command was applied to cluster the deduplicated sequences, and the otutab command was used to generate an OTU table. Then, we used Diamond (Buchfink et al., 2015) to construct a reference dataset from NCBI and perform alignments with the OTUs. Additionally, we identified the OTUs that failed offline identification in the online NCBI database. The Taxonkit (Shen and Ren, 2021) was used to obtain the taxonomic ID of the aligned sequences. After species identification, filtering was carried out. OTUs with a percentage identity (Per. ident) greater than 99% were considered identified to the species level, and those with a Per. ident greater than 95% were considered identified to the genus level. Species not distributed in the study area were removed.

To quantify trophic niche differentiation between the SE and TE groups, three key niche indices were calculated based on OTU-level dietary composition data. Shannon-Wiener Index (H') reflects the evenness of resource use. Levins' Index (B) measures the breadth of resource use. Pianka's Index confirms complete resource segregation and this index ranges from 0 (complete resource segregation) to 1 (complete overlap). All calculations were performed using the nicheROVER package in R. Visualization analysis was conducted using the R ggplot2 (Wilkinson, 2011) and the OmicStudio tools (Lyu et al., 2023) (https://www.omicstudio.cn/tool).

### 2.4 Metagenomics sequencing

Metagenomic sequencing of the stomach and gut microbiota from all samples was carried out by Novogene Co., Ltd. To obtain the clean data for subsequent analysis, the raw data from the Illumina sequencing platform were processed by removing the reads that contained low-quality bases more than a certain percentage (default: 40 bp); the reads with ≥10 bp N bases; and the reads whose overlap with the adapter exceeded a certain threshold (default:15 bp). To remove host contamination, we compared the sequencing data with the reference genome sequences from NCBI database. Specifically, we used the genome of *Sorex araneus* (GCF_000181275.1) for the SE group and the genome of *Rattus rattus* (GCF_011064425.1) for the TE group to filter out the reads that might originate from the host. The metagenomic data was assembled using Megahit (Li et al., 2015) and the assembled sequences were evaluated using Quast (Gurevich et al., 2013). The gene prediction was performed by Prodigal (Hyatt et al., 2010).

### 2.5 Species and function annotation

Taxonomy annotation was performed using Kraken2 (Wood et al., 2019, p. 2). Gene counts per species and family were merged via MetaPhlAn2 (Truong et al., 2015) to generate a gene catalog for α and β diversity analysis. Genes with ≥95% sequence identity and ≥90% coverage were clustered using CD-HIT (Fu et al., 2012), with representative sequences (longest from each cluster) used to build non-redundant gene catalogs. Gene abundance was quantified using Salmon (Patro et al., 2017), with per-sample abundances merged via Salmon quantmerge. Functional annotation of non-redundant gene catalogs used multiple methods: Diamond (Buchfink et al., 2015) aligned genes to five databases: Carbohydrate-Active Enzymes Database (CAZy) (Cantarel et al., 2009), Pathogen Host Interactions Database (PHI) (Urban et al., 2020), Virulence Factors Database (VFDB) (Chen et al., 2005), Comprehensive Antibiotic Resistance Database (CARD) (Alcock et al., 2020), eggNOG database (Huerta-Cepas et al., 2019). KEGG homologs (KOs) were annotated using Kofamscan (Aramaki et al., 2020).

### 2.6 Reconstruction of metagenome-assembled genomes (MAGs)

To retrieve low-abundance MAGs as much as possible, co-assembly of three batches which was stomach samples of SE group, gut samples of SE group, and all samples of TE group were separately performed using Megahit (Li et al., 2015). Binning of metagenomes was performed by three methods in Metawrap (Uritskiy et al., 2018): metabat2, maxbin2, and concoct. The bin_refinement module in Metawrap was used to refine the bin. The dRep dereplicate module was used to filter the refined bins. The classify_wf module in Gtdbtk (Chaumeil et al., 2020) was used for species annotation. Sequences of MAGs were aligned using Muscle (Edgar, 2004) and then phylogenetic tree was constructed by Raxml (Silvestro and Michalak, 2012).

## 3 Results

### 3.1 Dietary characteristics of the web-footed shrew reveal their specialized adaptation to river ecosystems

**Figure 2:**
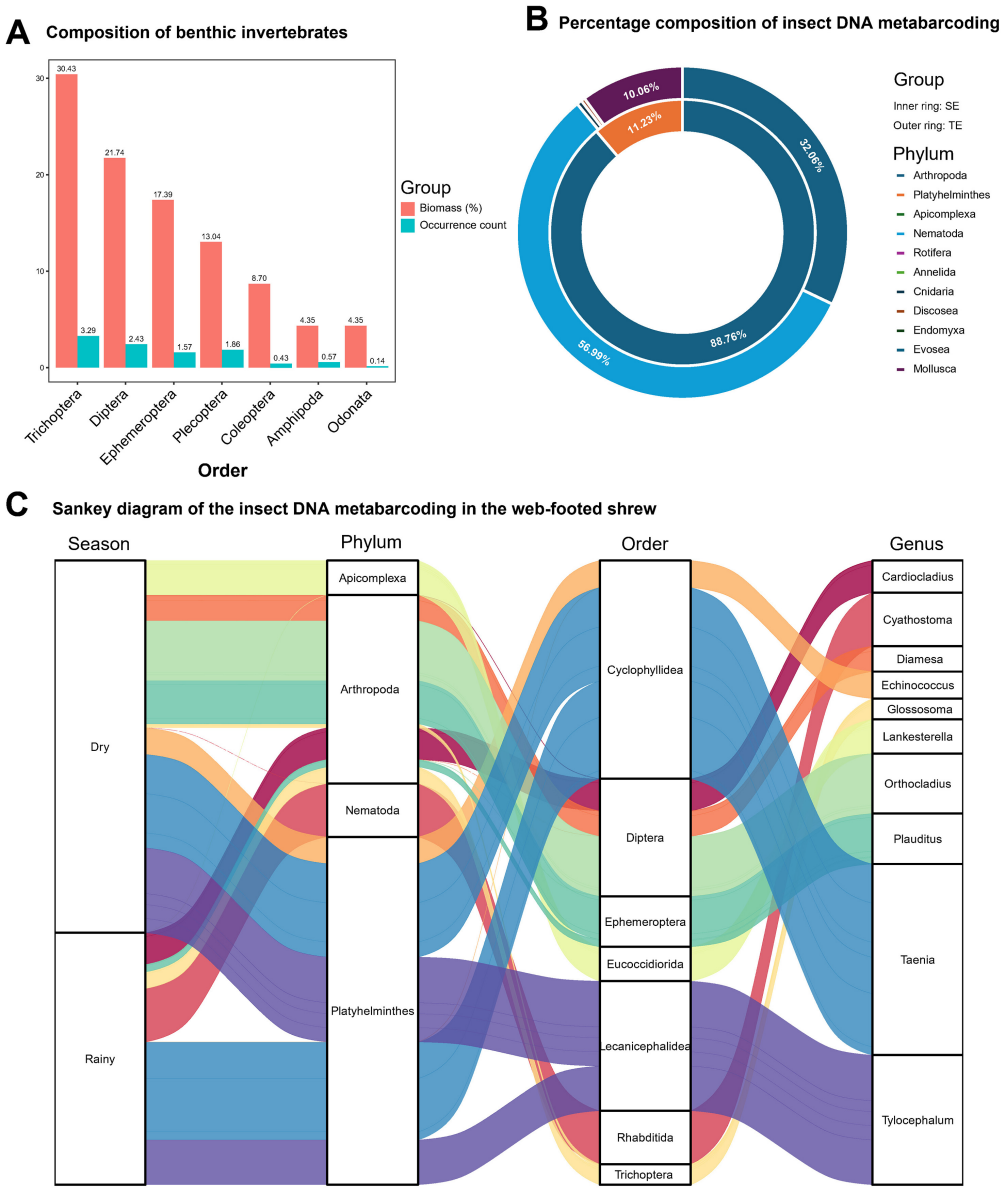
Food spectrum analysis. **(A)** Visual identification of benthic invertebrates: biomass (%) and Occurrence count at the Order level. **(B)** Percentage composition at the phylum level of the insect DNA metabarcoding in SE and TE. **(C)** Sankey diagram of the insect DNA metabarcoding of the web-footed shrew at diffrent level in the dry and rainy season.

This study investigated the community composition of benthic macroinvertebrates in sampled rivers during both dry and rainy seasons. Field surveys identified 1 class, 7 orders, 18 families, and 18 genera of benthic macroinvertebrates (Supplementary Table 3). In biomass, the top three orders were Trichoptera (30.43%), Diptera (21.74%), and Ephemeroptera (17.39%) (Figure 2A). Most morphological identifications were conducted at the genus level, with only 7 species identified to the species level (Supplementary Table 3). Seasonal variation analysis revealed no significant differences in benthic macroinvertebrate biomass between seasons (*P* > 0.05). Dietary DNA metabarcoding of the web-footed shrew showed that animal-based food primarily consisted of invertebrates with minor fish components. Based on COI metabarcoding data, the insectivorous diet of the shrew relied heavily on Diptera (44.40%), Ephemeroptera (29.22%), and Trichoptera (14.94%) within the phylum Arthropoda, directly corresponding to the top three biomass orders in benthic communities (Figure 2B). Dominant genera: *Heptagenia* (Heptageniidae, 22.96%), *Cricotopus* (Chironomoidea, 21.71%), *Glossosoma* (Glossosomatidae, 12.66%), and *Rheotanytarsus* (Chironomoidea, 9.25%) (Figure 2C), reflected specialized strategies for exploiting larvae in semi-aquatic habitats. The high protein larvae meet high metabolic demands and nutritional requirements of the shrew, forming feeding characteristics that are adapted to the dynamic changes of the river ecosystem. Analysis of 16S metabarcoding data detected trace amounts of Cypriniformes sequences, including *Schizothorax dolichonema, Schizothorax kozlovi, Schizothorax prenanti*, and *Gobio gobio* (Supplementary Table 4). The intake of fish components reflects the broad spectrum of their feeding habits and may serve as an energy supplementation mechanism. The seasonal stability of benthic biomass (*P* > 0.05) and the consistent dietary α-diversity of shrews (*P* > 0.05) indicate that stable food resources in this region maintain nutritional intake stability for the shrew.

### 3.2 Dietary divergence between the web-footed shrew and terrestrial rodents in sympatric distribution

Niche partitioning between the web-footed shrew and sympatric terrestrial rodents were achieved through dietary specialization. The web-footed shrew mainly feed on aquatic invertebrates with high energy density to meet their high metabolic demands. In contrast, dietary analysis of sympatric terrestrial rodents revealed a dual food resource strategy: animal-based diets were dominated by phylum Nematoda (56.99%), Arthropoda (32.06%), and Mollusca (10.06%) (Figure 2B), and plant consumption was primarily derived from the phylum Streptophyta, with the class Magnoliopsida (dicotyledonous plants) accounting for 99.47% of the total plant intake (Supplementary Table 4). The utilization of the *Physalis* plants (Solanales, 65.81%) by the terrestrial rodents reflects the utilization strategy of carbohydrate resources in the terrestrial environment. Terrestrial rodents mainly consume terrestrial nematodes and dicotyledonous plants, reflecting generalist feeding strategy.

### 3.3 Quantitative analysis of niche differentiation

**Figure 3:**
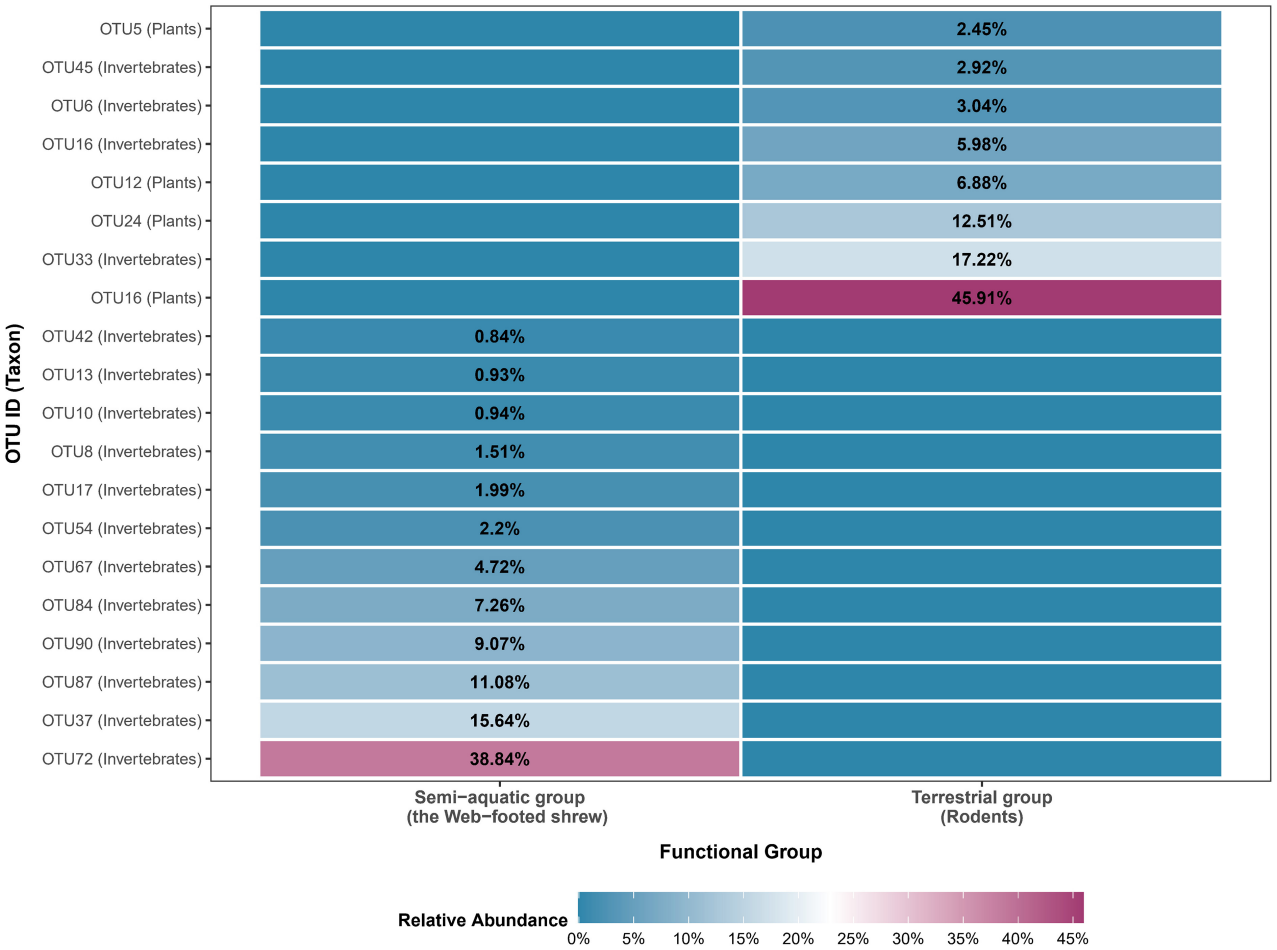
Heatmap of relative abundance of the top 20 dietary OTUs.

Niche width and overlap indices revealed significant trophic differentiation between the SE and TE groups, with complete segregation of core resources (Figure 3). The SE group exhibited a slightly higher resource use evenness (Shannon-Wiener Index, H' = 2.09) compared to the TE group (1.76). Similarly, the SE group had a higher resource use breadth (Levins' Index, B=4.88) than the TE group (3.75). Consistent with the Shannon-Wiener results, this confirms that SE shrews exploit a broader subset of available aquatic resources, while rodents in TE group are more specialized in exploiting limited terrestrial resources. A Pianka's Index of 0 indicated complete resource segregation. The shrews' diet was dominated by aquatic invertebrates, whereas rodents relied on terrestrial resources, with no overlap in core resource use. This pattern was visually supported by the heatmap of the top 20 OTUs (Figure 3), where aquatic-associated OTUs (e.g., Ephemeroptera, Trichoptera larvae) were exclusively detected in the SE group, while terrestrial-associated OTUs (e.g., Magnoliopsida plants) were restricted to the TE group, with no overlap in core resource utilization.

### 3.4 Distinctive gut microbial community structures between sympatric semi-quatic (SE group) and terrestrial (TE group) mammals

The metagenome sequence data encompassed a total of 684.95 Gb raw data, with an average of 19.57 Gb per sample. After size filtering and quality control, a total of 652.09 Gb clean data were obtained, which accounted for more than 95.42% of the raw data. The assembly of these clean reads resulted in a total of 27.02 million contigs (Supplementary Table 5). Based on these contigs, 19.33 million unigenes with an average length of 416 bp and an average GC content of 37.09% were obtained.

**Figure 4:**
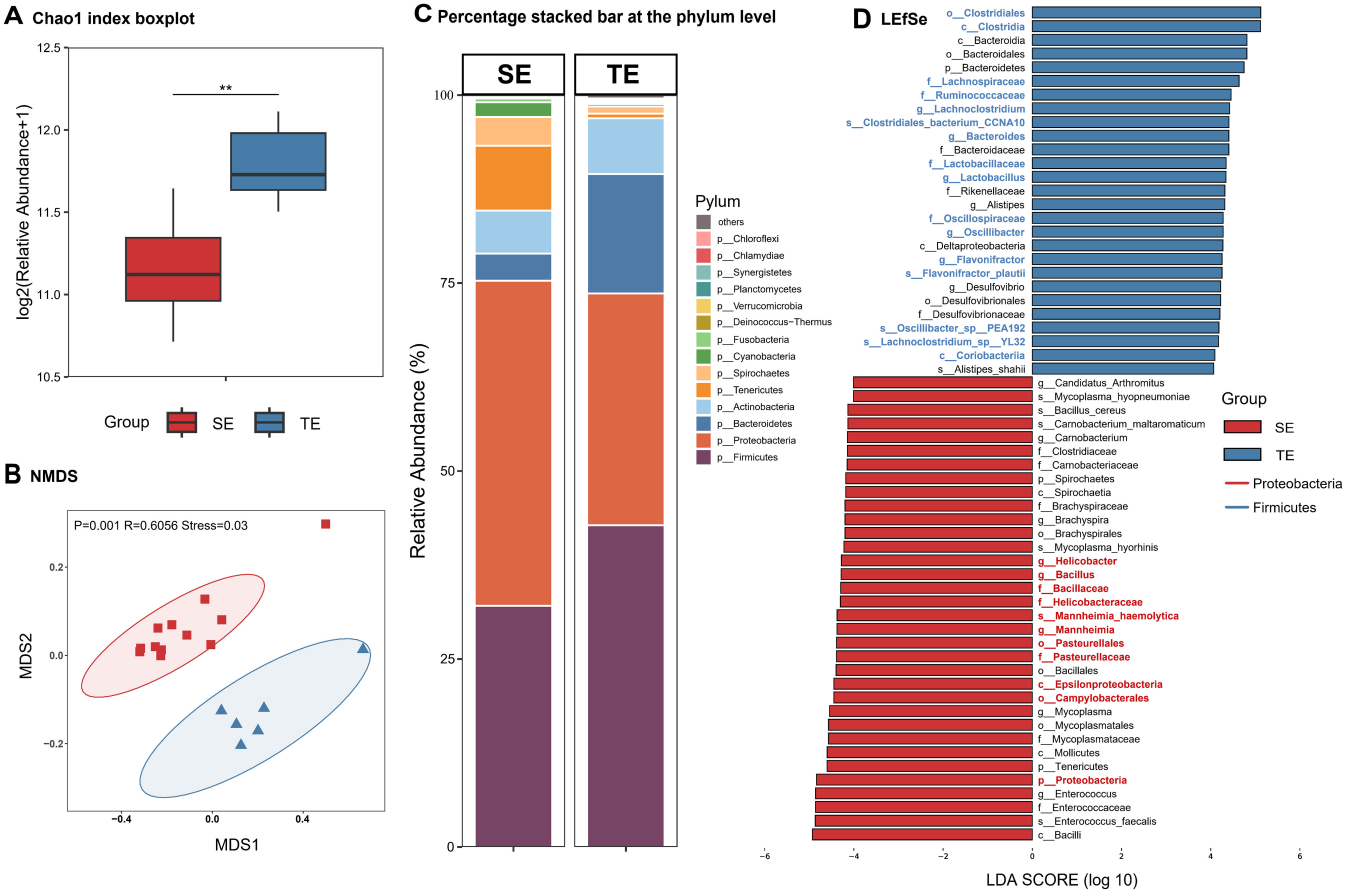
Differences in gut microbial composition between SE and TE. **(A)** Chao1 index. **(B)** NMDS analysis. **(C)** Percentage stacked bar at the phylum level. **(D)** LEfSe analysis (LDA > 4). *p < 0.05, **p < 0.01, ***p < 0.001.

The gut microbiota composition of the semi-aquatic web-footed shrews (SE group) and terrestrial rodents (TE group) in sympatric distribution exhibits marked divergence. Both α-diversity (Figure 4A) and β-diversity (Figure 4B) revealed significant structural differentiation (*P* < 0.01). SE group was dominated by phylum Proteobacteria (43.22%), significantly higher than TE group (32.09%), while Firmicutes abundance in TE group (42.78%) was elevated compared to SE group (30.83%) (Figure 4C). SE group harbored 34 unique microbial species, whereas TE group contained 41 specific taxa. LEfSe analysis identified 61 differentially abundant taxonomic groups (LDA > 4, Figure 4D), with 34 enriched in SE group (e.g., Proteobacteria-related clades) and 27 in TE group (e.g., Firmicutes-related clades).

### 3.5 Habitat-dependent divergence in gut microbial functions

**Figure 5:**
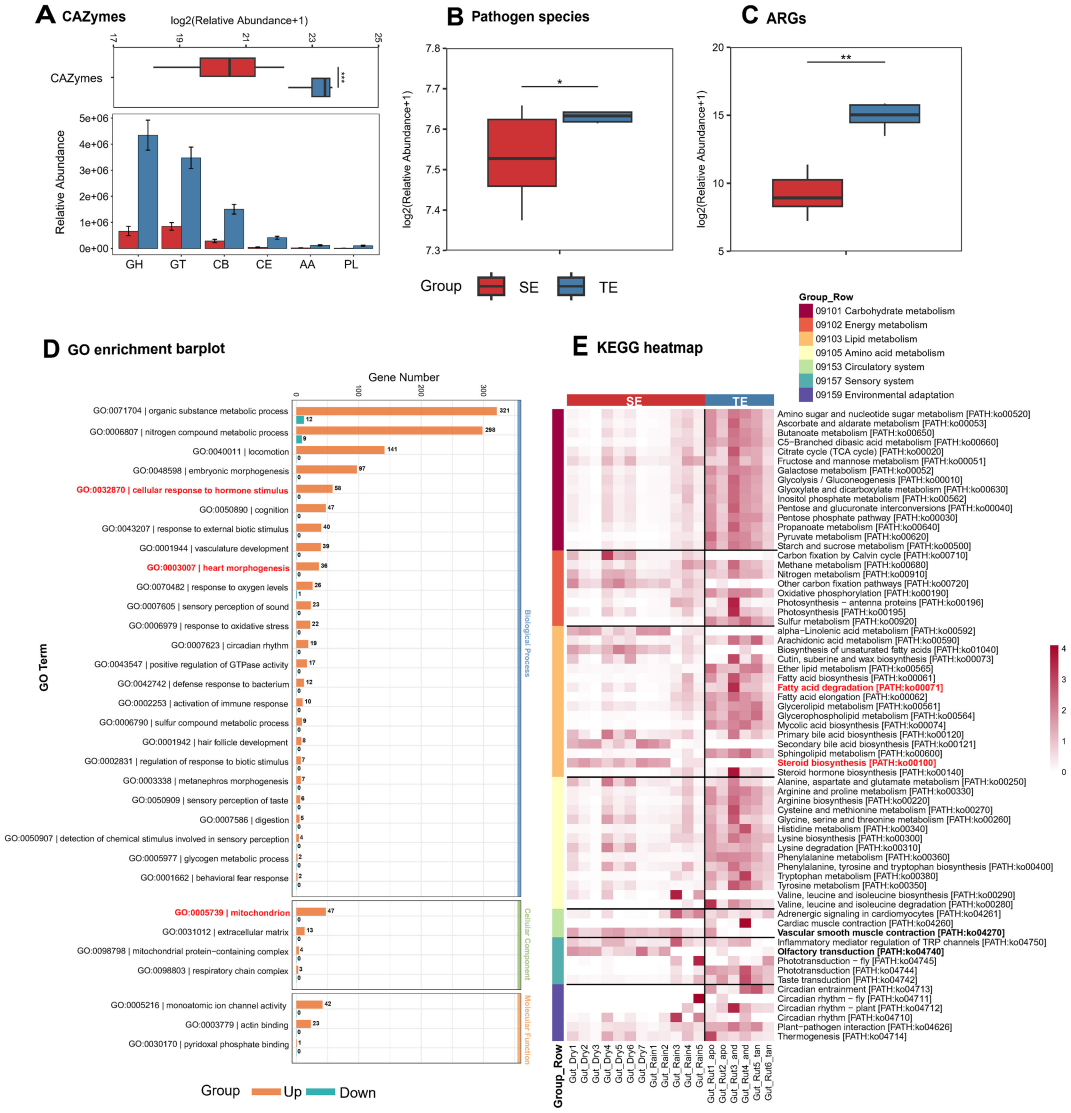
Differences in gut microbial functions between SE and TE. **(A)** Differences in CAZymes and a comparative analysis in the six CAZymes classes. **(B)** Differences in Pathogen species. **(C)** Differences in ARGs. **(D)** GO enrichment barplot associated with semi-aquatic adaptations. **(E)** KEGG heatmap of SE and TE. *p < 0.05, **p < 0.01, ***p < 0.001.

The gut microbiota of the web-footed shrew exhibits functional specialization aligned with their ecological niche. Carbohydrate-active enzymes (CAZymes) were significantly less abundant in SE group compared to TE group (Figure 5A), linked to their minimal carbohydrate intake (reliance on high-protein, high-fat aquatic insects). Pathogen species were more abundant in TE (Figure 5B), suggesting greater exposure to terrestrial pathogens. Antibiotic resistance genes (ARGs) in SE group were significantly lower than TE group (Figure 5C), possibly resulting from plant secondary metabolites or soil antibiotic pressures in terrestrial environments. These differences highlight divergent microbial adapt strategies shaped by dietary constraints and environmental stressors, underscoring microbiota adaptation to contrasting ecological niches.

KEGG and GO metabolic pathway divergence reveals coevolutionary strategies for ecological adaptation. KEGG pathway analyses indicate that carbohydrate metabolism (09101) in TE group is important for environmental adaptation (Figure 5E). In contrast, although the overall abundance of metabolic pathways in SE group is lower than that in TE group, the shrew preferentially activates energy and lipid metabolism to meet the demands of diving and low temperatures (Figure 5E). Circulatory system (09153) and sensory system (09157) pathways further enhanced aquatic physiological functions (e.g., blood flow regulation ko04270, chemical signal perception ko04270, Figure 5E, Supplementary Figure 1). Notably, selective upregulation of K00632/K00022/K01692 in fatty acid metabolism (ko00071) enabled efficient utilization of high-fat diets (Supplementary Figure 2) and enriched bile acid metabolism (ko00100) pathways improved lipid utilization efficiency (Supplementary Figure 3) demonstrating the precise enhancement of key survival pathways by the shrew. Additionally, the upregulation of mitochondrial function genes (GO:0005739) in the GO metabolic pathway (Figure 5D) enhanced the energy conversion from lipids. The upregulation of single-atom ion channel activity genes (GO:0005216) and salinity tolerance genes (GO:0006807) (Figure 5D) optimize the regulation of osmotic balance. The upregulation of brown adipose activation-related genes (GO:0032870) supports the maintenance of body temperature in cold water. In addition, the enrichment of genes related to cardiac morphogenesis (GO:0003007) synergistically enhances the support of the cardiovascular system for diving behavior (Figure 5D). These functional specializations together construct a microbe-host collaborative mechanism for adapting to the river ecosystem of the shrew.

### 3.6 Contrasting characteristics of gastrointestinal microbiota in the web-footed shrew

**Figure 6:**
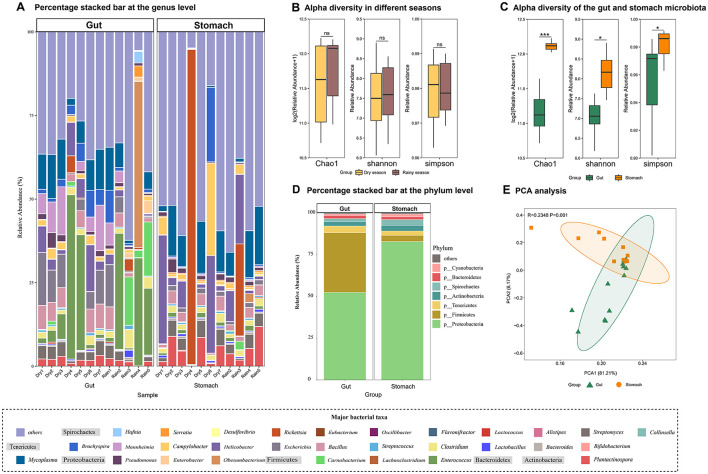
Characteristics of the gut and stomach microbiota and differences in gut microbial composition in dry season and the rainy season of the web-footed shrew. **(A)** Percentage stacked bar of the gut and stomach microbiota at the genus level. **(B)** Alpha diversity of the gut microbiota in the dry season and the rainy season. **(C)** Alpha diversity of the gut and stomach microbiota. **(D)** Percentage stacked bar of the gut and stomach microbiota at the phylum level. **(E)** CA analysis of the stomach and gut microbiota.

The stomach and gut microbiota of the web-footed shrews exhibit synergistic adaptation to high-protein diets and semi-aquatic habitats through functional partitioning and structural divergence. Stomach microbiota are dominated by Proteobacteria (82.92%, Figure 6D), with specialized enrichment of acid-tolerant taxa such as *Helicobacter* (Figure 6A). Gut microbiota form a complementary structure of Proteobacteria (52.47%) and Firmicutes (35.75%) (Figure 6D), with genera like *Escherichia* (Proteobacteria) and *Enterococcus* (Firmicutes) enhancing amino acid metabolism and energy absorption (Figure 6A). Chao1, Shannon, and Simpson index of stomach microbiota were all significantly higher than gut microbiota (Figures 6C, E), and the high diversity of stomach microbiota may compensate for the need to decompose complex foods, while the low diversity of the gut microbiota improves the efficiency of nutrient utilization through functional specificity. This zonal specialization is highly consistent with the host's digestive physiology (a strongly acidic stomach environment vs. a neutral intestinal environment). Moreover, the differences in unique species between the stomach (774 species) and the gut (71 species) further optimizes the functional connection of the digestive chain. Moreover, the seasonal stability of the microbial community structure (*P* > 0.05, Figure 6B) works in synergy with the steady supply of food biomass. These findings highlight microbiota specialization driven by dietary divergence and environmental pressures (aquatic vs. terrestrial feeding), reflecting adaptive responses to distinct ecological niches.

### 3.7 Reconstruction of metagenome-assembled genomes (MAGs) from metagenomic datasets

**Figure 7:**
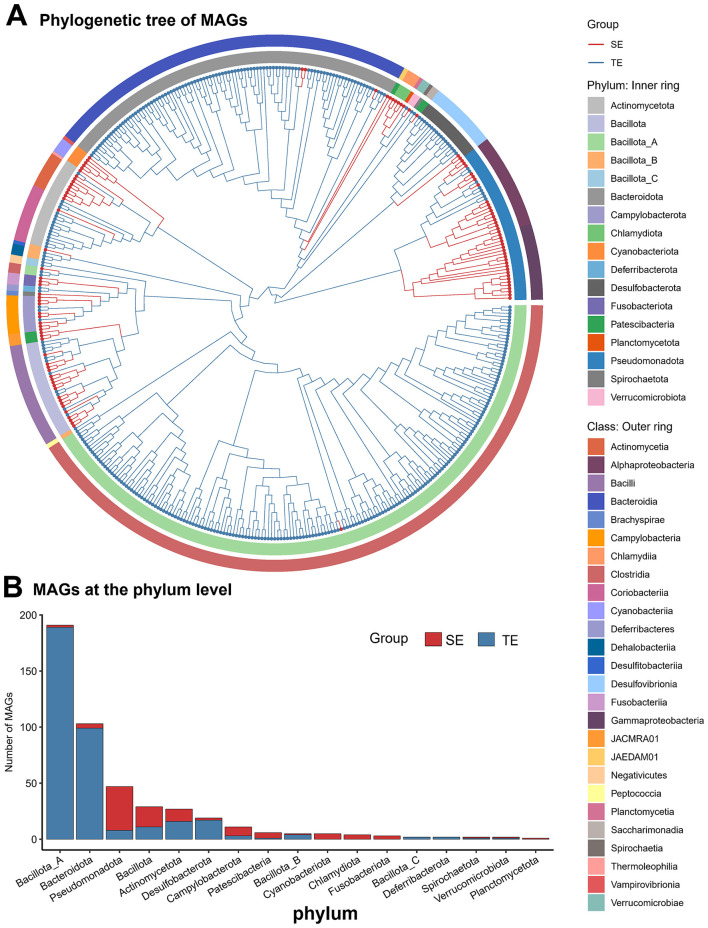
Metagenome-assembled genomes (MAGs). **(A)** Phylogenetic tree of MAGs from SE and TE. **(B)** The number of MAGs from SE and TE at the phylum level.

The MAGs in the web-footed shrew were dominated by Proteobacteria (Currently: Pseudomonadota) and Firmicutes (Currently: Bacillota), showing significant differences from the Firmicutes_A (Currently: Bacillota_A) and Bacteroidetes (Currently: Bacteroidota) profiles observed in terrestrial rodents (Figure 7B). This compositional difference may reflect diet-driven niche differentiation: the enrichment of Proteobacteria in the SE group may be related to the high protein demand of aquatic insects in their diet, while the dominance of Firmicutes_A in the TE group may be associated with the decomposition of plant fibers. The phylogenetic tree shows that the MAGs of SE and TE groups cluster separately (Figure 7A), further supporting the environmental-specific differentiation of the microbial communities. The unique MAGs in the SE group (Figure 7B) may be involved in adaptation to the aquatic environment. These compositional characteristics provide a microbial basis for SE group to occupy the river niche.

## 4 Discussion

Factors determining the composition and function of host-associated microbial communities are central to microbial ecology (Sadeghi et al., 2023). The roles of these microorganisms in facilitating vertebrate hosts' environmental adaptation have also attracted significant attention (Karasova et al., 2022). In this study, we compared sympatric species with distinct environmental adaptations to investigate the composition and interactions between host diets and gut microbial communities under fully natural conditions. Our data confirmed that environmental factors influence species' feeding preferences, and dietary composition shapes gut bacterial community structure. We further demonstrated that species with unique environmental adaptations can realize their functional potential while reducing competition with other sympatric species, providing evidence for the role of diet-gut microbiota interactions in mediating ecological adaptation.

The feeding habits of the web-footed shrew demonstrate a high degree of adaptation to river ecosystems, with their dietary composition closely aligned with benthic macroinvertebrate community structures. The semi-aquatic environment provides animals with abundant and diverse aquatic organisms as food sources (Soukup et al., 2022). In this study, samples of the potential foods of the shrew were obtained from field surveys, and the dietary composition was investigated based on DNA metabarcoding. It was found that the species mainly feed on benthic invertebrates and some freshwater fish species. The invertebrate diet mainly comprises Arthropoda phylum organisms, with dominant genera including *Plauditus, Cardiocladius, Glossosoma*, and *Diamesa*. The larvae of the above genus mostly prefer to inhabit water areas with relatively gentle water flow, clear water quality, and rich oxygen content (Wang et al., 2022). The freshwater fish mainly include species of the order Cypriniformes: *Schizothorax dolichonema, Schizothorax kozlovi, Schizothorax prenanti*, and *Gobio gobio*. These species are all distributed in the Yalong River Basin (Gao et al., 2024). These fish are excellent sources of high-quality protein and essential fatty acids, which are beneficial for the growth and development of the shrew (Shahzad, 2024). This energy supply helps the shrew to sustain its high metabolic rate, enabling it to adapt to the cold and challenging aquatic conditions and continue its daily survival behaviors (Glazier, 2024).

Quantitative niche indices confirm robust trophic segregation between SE and TE groups, revealing adaptive strategies tailored to the arid-hot valley ecosystem. The higher Shannon-Wiener index of SE group (H' index: SE = 2.09, TE = 1.76) reflects more even use of aquatic invertebrates, enabled by stable, diverse benthic communities (e.g., Ephemeroptera, Trichoptera larvae) in valley streams. In contrast, the TE group exhibits a lower Shannon-Wiener index, which stems from its reliance on a narrow set of core terrestrial resources. The lower evenness reflects an adaptation to the valley's fragmented, resource-fluctuating terrestrial environment, where drought-tolerant plants and nematodes serve as reliable food sources. Consistent with this, the higher Levins' index of SE group (B index: SE = 4.88, TE = 3.75) indicates broader exploitation of aquatic resources, while TE's narrower breadth reflects a trade-off: focusing on limited terrestrial resources reduces foraging costs in patchy, variable habitats. A Pianka's index of 0 confirms complete resource segregation between the two groups: the SE group relies on aquatic invertebrates, while the TE group depends on terrestrial resources. This strict resource partitioning eliminates interspecific competition for core food sources. This pattern aligns with niche theory, where sympatric species minimize competition through resource partitioning. Here, this partitioning is driven by their distinct microhabitat preferences. These results quantify the observed trophic differentiation, highlighting specialized strategies that support coexistence in the arid-hot valley.

Dietary specialization enabled niche partitioning between the web-footed shrew and its sympatric terrestrial rodents. The shrew primarily consumes animal-based diets, whereas terrestrial rodents exhibit omnivorous feeding habits, incorporating both animal and plant resources. These results align with previous research (Peng and Tao, 2023). This dietary specialization reflects differences in food diversity between semi-aquatic and terrestrial environments and highlights habitat-specific patterns in food composition and structure. More specifically, the insect-based feeding strategy of the shrew confers significant adaptive advantages for its survival and reproduction. Benthic insects, as a rich and stable food resource, enable the shrew to occupy a unique ecological niche, thereby reducing competition with other sympatric species (Charbonnel et al., 2015). Members of the Soricidae family exhibit extremely high metabolic rates, a physiological trait closely linked to their feeding habits (Holland, 2024). The shrew's high metabolic demands necessitate a large energy supply. Insects, rich in proteins and fats, efficiently meet these energy requirements and support normal growth, development, and reproduction [66,67]. Insects' relatively simple body structures, composed of chitinous exoskeletons and internal soft tissues (Chapman, 1971), allow rapid decomposition and absorption in the shrew's digestive system (Kim et al., 2023). Shrews possess a specialized insectivorous digestive tract that is adapted to processing high-protein diets, facilitating efficient energy absorption and conversion (McCay and Storm, 1997; Ochocińska and Taylor, 2005; Karasov and Douglas, 2013). In contrast, plant-based foods provide lower energy density, making them inadequate for meeting the shrew's extreme energy demands (Williams and Kay, 2001). Generalist foraging patterns are demonstrated by terrestrial rodents through their consumption of both terrestrial nematodes and dicotyledonous plant materials. The habitat segregation in diet (aquatic vs. terrestrial) and the differences in trophic level (secondary consumers vs. primary consumers) between the two groups effectively reduce resource competition (Sun et al., 2019). This co-evolutionary niche partitioning mechanism promotes the coexistence of species and the efficient distribution of energy in the ecosystem.

Marked divergence can be observed in the gut microbiota composition of sympatric semi-aquatic web-footed shrews and terrestrial rodents. Results demonstrate significant differences in gut microbial communities between the two groups at the phylum level (Proteobacteria vs. Firmicutes) and in diversity indices (α/β diversity, *P* < 0.01). Semi-aquatic shrew exhibits a unique microbial community dominated by Proteobacteria (aerobic bacteria), whereas terrestrial mammals show enrichment of Firmicutes (predominantly anaerobic bacteria) (Bletz et al., 2016). This pattern is closely linked to dissolved oxygen levels in their respective habitats (Waters, 1996): the semi-aquatic environment maintains high oxygen concentrations through water turbulence, favoring colonization by aerobic Proteobacteria, while the anaerobic gastrointestinal microenvironment of terrestrial animals supports metabolic activities of anaerobic Firmicutes. The elevated abundance of Proteobacteria in SE group may relate to their diet of chitin-rich aquatic insects, as these bacteria potentially assist digestion via chitinase secretion (Bletz et al., 2016). The niche construction theory emphasizes how organisms actively shape environments to enhance fitness. In our research, the niche construction theory is reflected in two aspects: the web-footed shrews specialize in preying on high-energy aquatic invertebrates, which not only adapts them to semi aquatic ecological niches, but may reduce benthic prey competition for sympatric aquatic organisms. Meanwhile, the enhanced fatty acid metabolism of the gut microbiome further shapes the ability of shrews to utilize aquatic resources, forming positive feedback between niche construction and microbial adaptation. Conversely, Firmicutes in TE group likely generate short-chain fatty acids through fermentation of terrestrial plant fibers, providing supplementary energy for hosts (Kostopoulou et al., 2023). These differences highlight the direct impact of environmental selective pressures on gut microbiota composition, reflecting co-evolutionary strategies in oxygen utilization between hosts and microbes.

Under environmental stress, the balance between dietary stability and microbial diversity promotes species coexistence (Moeller and Sanders, 2020). The reduced species richness (Chao1 index) in gut microbiota of the web-footed shrew correlates with their stable diet of benthic aquatic insects (Reese and Dunn, 2018). Seasonal stability of food resources in semi-aquatic ecosystems (*P* > 0.05) likely ensures energetic metabolic efficiency by minimizing microbial fluctuations (Costa and Favilla, 2023). This positive feedback loop between stable diet and microbial structure supports continuous energy metabolism adapted to riverine ecosystem demands. In contrast, TE group requires more complex microbial communities to degrade diverse nutrients from omnivorous diets, maintaining higher species richness (Lavrinienko et al., 2018). These findings align with the “niche allocation efficiency” theory (Meier et al., 2023). SE group enhances utilization efficiency of singular food resources through specialized microbiota structuring, while TE group relies on diverse microbiota to accommodate complex dietary requirements (Maritan et al., 2024).

Functional gene differences directly reflect differential environmental pressures experienced by hosts (Zhang et al., 2025). Microbiota of the web-footed shrew maximizes energetic metabolic efficiency in resource-stable aquatic environments through reducing non-essential functional genes (e.g., CAZymes) and strengthening key metabolic pathways (e.g., fatty acid metabolism), aligning with the “reduced diversity but enhanced functional specialization” niche optimization strategy (Xiong et al., 2024). Additionally, SE group displayed far lower antibiotic resistance genes (ARGs) abundances compared to TE group, likely due to reduced antibiotic exposure in aquatic habitats, whereas the enrichment of ARGs and PHI genes in TE group suggests long-term adaptation to plant defense compounds or soil microbial competition in terrestrial environments, where mammals face greater antibiotic and pathogen challenges (Wang et al., 2024). KEGG pathway analysis reveals niche-specific optimization of host energy demands through divergent metabolic strategies: SE group preferentially activates lipid metabolism to meet diving and cold exposure requirements (e.g., high expression of fatty acid key genes), whereas TE group strengthens carbohydrate metabolism to support omnivorous dietary demands (Chen et al., 2023). The streamlined GO carbohydrate metabolism-related genes in gut microbiota of the web-footed shrew may represent an energy-saving strategy prioritizing resource allocation to energy and lipid metabolic pathways. Microbiota and host gene expression of SE group form a synergistic network to address aquatic environmental challenges, including energy metabolism regulation, thermoregulation, and diving behavior support. Specifically, the upregulation of microbial fatty acid metabolism genes and host mitochondrial function genes (GO:0005739) optimizes lipid utilization efficiency in cold water (Zhang et al., 2022). Upregulation of brown adipose activation genes (GO:0032870) supports thermogenesis under low temperatures (Wang C. H. et al., 2023), and enhanced cardiomyocyte morphogenesis genes (GO:0003007) cooperatively improve cardiovascular blood flow regulation during diving (Gu et al., 2022).

The gastrointestinal microbiota of the web-footed shrew achieves efficient adaptation to high-protein diets and semi-aquatic environments through three key mechanisms: functional partitioning in synergy with host digestive physiology, balances between microbial diversity and functional efficiency, and life-driven microbial community dynamics. In this study, semi-aquatic lifestyles dynamically shape gastrointestinal microbial communities. The stomach microbiota, dominated by Proteobacteria, exhibits specialized acid-tolerant bacteria (MAGs of Proteobacteria) that not only tolerate acidic environments but also initiate chitin degradation from insect exoskeletons via secreted chitinases (e.g., GH18 family) (Cao et al., 2022). This functional specialization, in synergy with pepsin, breaks down insect fats and proteins into oligopeptides, laying the foundation for subsequent gut absorption (Daniel et al., 2014). The complementary structure of Proteobacteria and Firmicutes in the gut significantly enhances host energy absorption efficiency for high-protein diets (Cai et al., 2022). The stomach's high diversity compensates for complex decomposition demands, while the gut's low diversity optimizes specialized functional efficiency. This differential richness reflects niche-specific functional requirements. Semi-aquatic lifestyles may influence gastrointestinal microbial distribution through two mechanisms: feeding behavior dilution effects and high-frequency foraging effects. Aquatic foraging results in copious water intake during each meal, diluting stomach juices and promoting colonization by novel microbes (e.g., environmental Proteobacteria), forming a dynamically diverse stomach microbiota. High-frequency feeding generates complex stomach contents, where metabolically redundant high-diversity microbes buffer dietary fluctuations to ensure effective degradation of fat and proteins (Cao et al., 2022). Frequent feeding also dilutes stomach juices and promotes microbial turnover, stabilizing intestinal environments to support functional specialization. The gut's stable neutral environment allows specialized microbial consortia to efficiently execute amino acid absorption and energy conversion (Li et al., 2024). This compartmental strategy, characterized by “stomach priming-gut deepening” digestive chain division of labor, aligns seamlessly with host digestive physiology (acidic stomach vs. neutral gut) to optimize functional continuity along the digestive tract (Meier et al., 2023).

The diet and microbiota of the web-footed shrew in the dry-hot valleys of the Hengduan Mountains showed no significant seasonal variation between dry and wet seasons, may attributed to hydrological stability maintained by alpine snowmelt, groundwater recharge, and water temperature buffering, combined with continuous benthic invertebrate food availability (Cao et al., 2015). A comparative analysis of the field surveys, feeding habits and gut microbiota of the two seasonal samples revealed that there were no significant seasonal changes of the shrew in the study site. The sampling site is in a tributary of the Yalong River in the Hengduan Mountains, which is in the dry-hot valley area. This tributary has a stable water source supply, mainly from precipitation, alpine snowmelt and groundwater. In the dry season, although the precipitation decreases, the continuous supply of alpine snowmelt and groundwater maintains the streamflow, preventing the river from drying up. And due to the large specific heat capacity of water, the increase rate of the land surface temperature is higher than that of the water body, and the water environment temperature is more stable than the land environment temperature (Byrne and O'Gorman, 2013). Combined with the results of the morphological identification of benthic invertebrates at the sampling site, it can be inferred that the water ecosystem in this area is relatively stable.

This study employed DNA barcoding to analyze the composition of the feeding habits. Nevertheless, the sequencing results of this method contain a large amount of host DNA sequences, especially in the 16S barcode results of vertebrates, which are contaminated by massive host DNA sequences. Future research can utilize blocking primers to prevent the massive amplification of host DNA (Reigel et al., 2020) and to study diet composition more accurately and comprehensively. In addition, in the sample selection of this study, the web-footed shrew was chosen to be compared with rodents instead of other shrews. The reason is that in the low-altitude areas near the rivers in the dry-hot valley region, terrestrial shrews are extremely scarce, making it difficult to capture individuals with sympatric distribution. Although there are other shrews in the mountain top forest areas, they have significant differences in altitude and habitat from the web-footed shrew, which does not conform to the concept of sympatric distribution. In the future, the frequency and duration of investigations can be increased, and diverse capture methods can be adopted to improve the probability of capturing terrestrial shrews. An alternative approach could be to change the study site to one where both terrestrial shrews and the web-footed shrew are present.

In the end, we assembled microbiome genome in the stomach and gut of the web-footed shrew at the genomic level, which provides a new perspective for better understanding the structure and function of the gut microbial community and the interaction mechanisms between microbes and hosts in semi-aquatic mammals. In future research, the roles of these microbiomes in semi-aquatic adaptation can be further investigated through bacterial culture. We have illustrated the adaptive characteristics of the web-footed shrew (*Nectogale elegans*) from multiple perspectives to determine how it can be most effectively used as a paradigm for research on the ecological adaptability of semi-aquatic animals. This has potential implications for a deeper understanding of the manner in which hosts adapt to changing environments in a broader context.

## 5 Conclusion

Using the web-footed shrew (*Nectogale elegans*) as a model, we've made key discoveries. Using benthic surveys and dietary DNA metabarcoding, we demonstrate that semi-aquatic specialization drives a protein-rich diet of benthic invertebrates and fish, supporting high metabolic demands. We've determined its diet, mainly Diptera, Ephemeroptera, and Trichoptera larvae, and found that it matches benthic biomass. This diet is an adaptation for accessing high—protein resources in rivers. By comparing with sympatric terrestrial rodents, we found the shrew differentiates its niche through habitat and trophic level divergence. Gut microbiome analysis shows significant differences between the sympatric shrew and rodents. The shrew's gut microbiome is characterized by unique features such as reduced abundance of certain genes related to carbohydrate metabolism and pathogen—host interaction, but enhanced functions in fatty acid and bile acid metabolism pathways. These reflect its adaptation to a high-protein, lipid-rich diet and semi-aquatic environmental pressures. Moreover, the microbiome of the shrew optimizes energy conversion, thermoregulation, and diving behavior in cold water. Our research not only provides a detailed understanding of the semi—aquatic adaptation mechanism of the web—footed shrew but also offers new insights into the ecological niche differentiation and specialization mechanisms in small mammals.

## Data Availability

The data presented in this study are publicly available. This data can be found here: https://www.ncbi.nlm.nih.gov/bioproject, accession PRJNA1322107.
