# Supplementary material for: Diet-microbiome coevolution: the core mechanism for semi-aquatic adaptation and cross-habitat niche coexistence of the web-footed shrew (Nectogale elegans)
Source: Front Microbiol. 2025 Nov 13;16:1711143. doi: 10.3389/fmicb.2025.1711143 (PMC12659910; doi:10.3389/fmicb.2025.1711143)
Supplement: Supplementary file 2 [file Data_Sheet_1.docx]

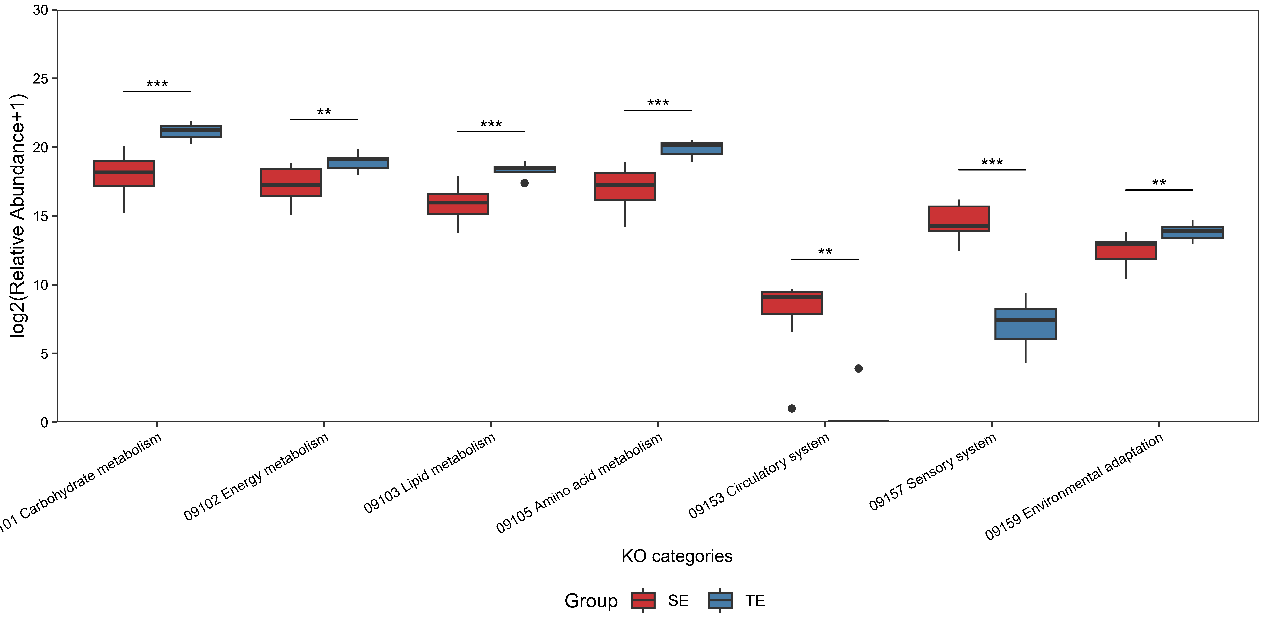


Supplementary Figure 1. Comparative analysis of the KO categories.


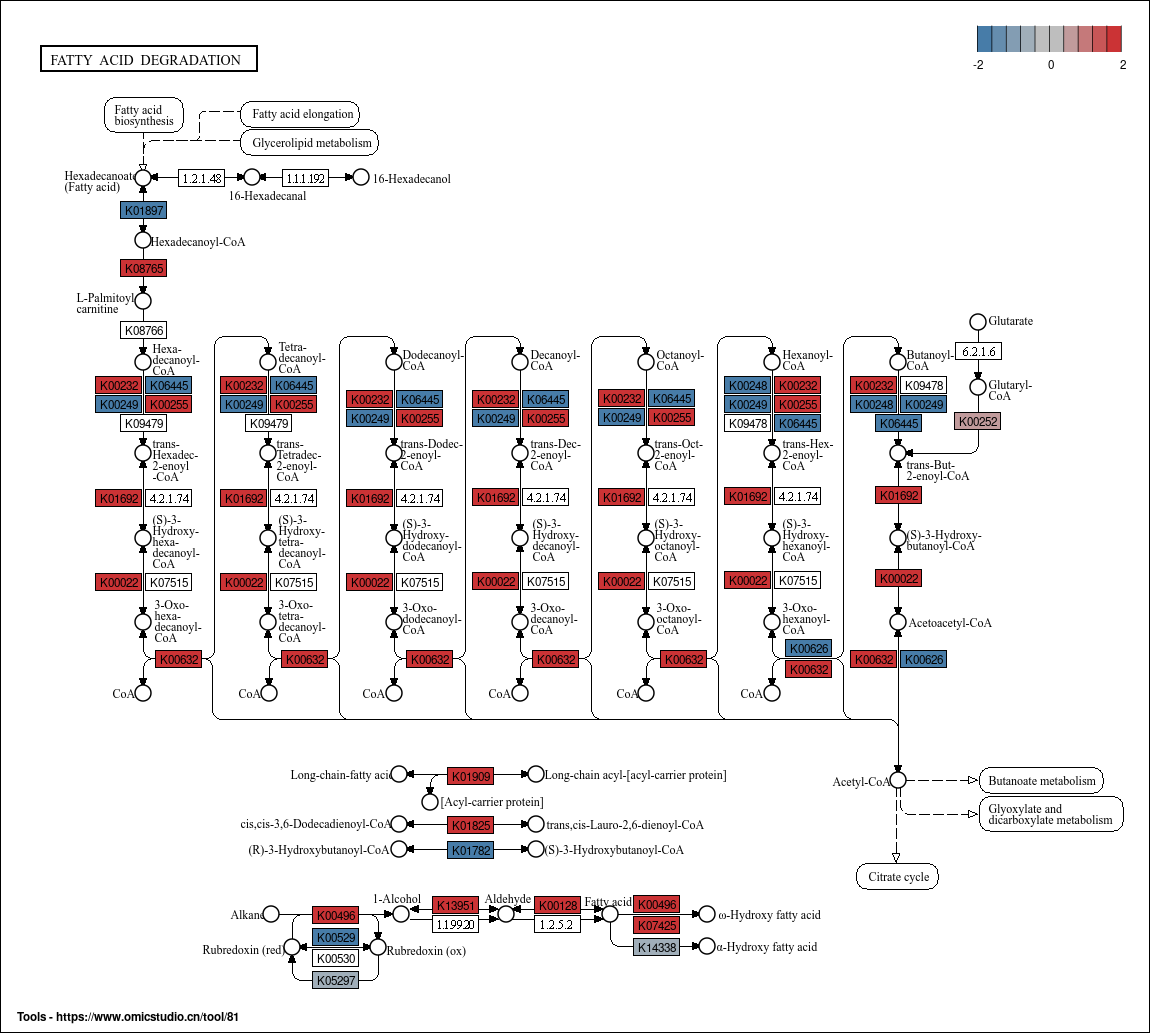


Supplementary Figure 2. Gene enrichment of fatty acid degradation (ko00071).


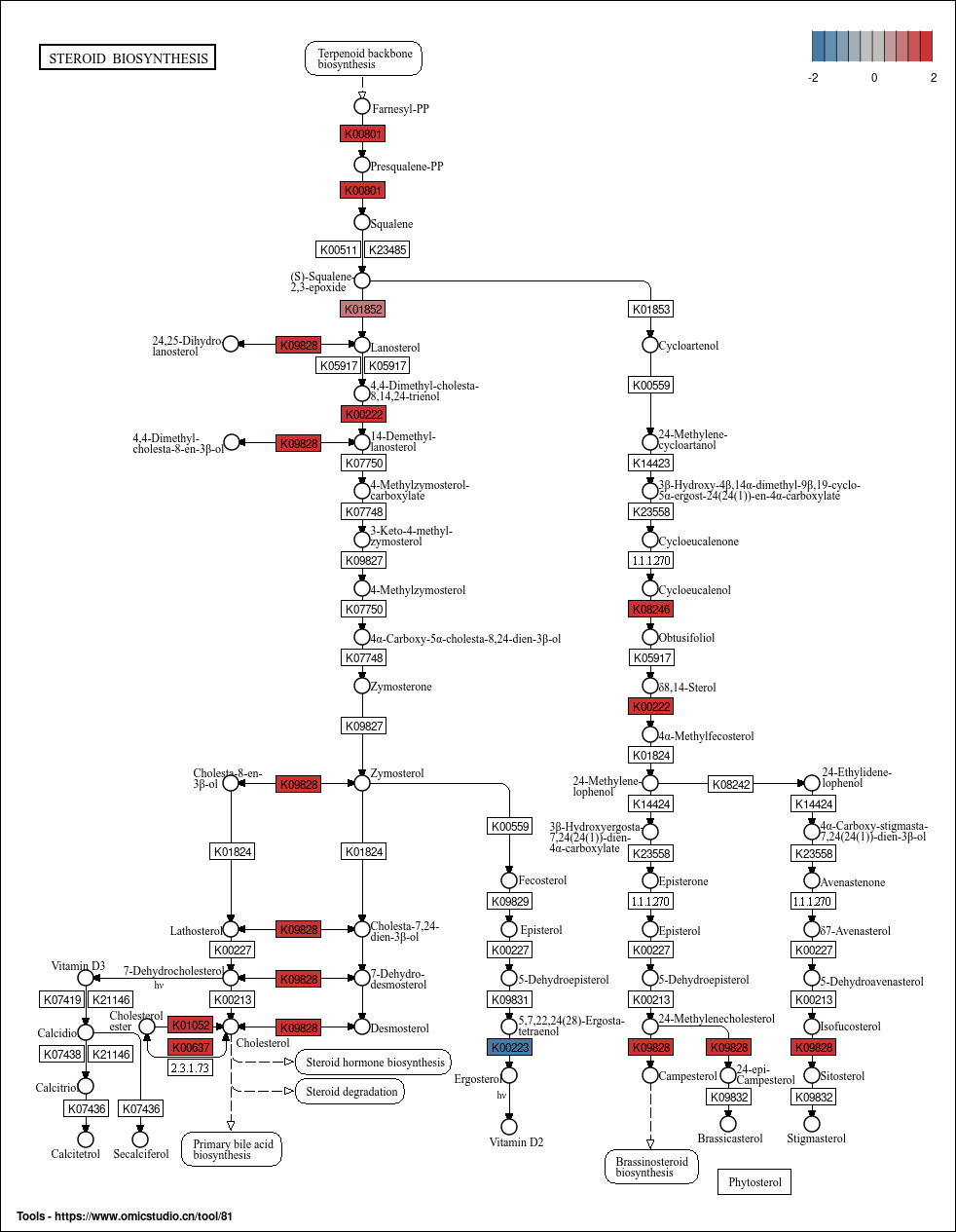
Supplementary Figure 3. Gene enrichment of steroid biosynthesis (ko00100).
